# Tunable Optical Bistability Within the Bandgap in One-Dimensional Photonic Crystal Multilayer Structures Containing Weyl Semimetal

**DOI:** 10.3390/mi17080986

**Published:** 2026-08-21

**Authors:** Liuxin Qian, Zhiheng Li, Zean Shen, Jiao Tang, Leyong Jiang

**Affiliations:** School of Physics and Electronics, Hunan Normal University, Changsha 410081, China

**Keywords:** optical bistability, Weyl semimetal, one-dimensional photonic crystal

## Abstract

In this paper, we propose a layered structure composed of Weyl semimetal (WSM) and one-dimensional photonic crystal (1D-PhC) to achieve low-threshold and tunable optical bistability (OB). By exploiting the bandgap characteristics of the photonic crystal and the strong third-order nonlinearity of WSM in the terahertz regime, reflective OB is realized within the photonic bandgap. It is shown that at certain frequencies inside the bandgap, the reflectance exhibits a jump with an increasing incident electric field, manifesting a clear hysteresis loop. The bistable threshold and hysteresis width can be effectively tailored by adjusting the Fermi energy and the thickness of the WSM layer. In addition, the influence of the incident angle and the parameters of the spacer layer on the OB threshold has also been elucidated. After parameter optimization, the incident electric field threshold is reduced to the order of 10^6^ V/m. We believe that this structure can provide a reference for constructing optical bistable schemes with simple structures and low thresholds.

## 1. Introduction

Optical bistability (OB) refers to the existence of two mutually switchable stable output states for a given input state in nonlinear optical systems, with the input–output characteristic curve exhibiting a typical hysteresis loop [1]. OB holds broad application prospects in micro-nano photonic devices, such as all-optical switches [2], optical transistors [3], optical memories [4], and all-optical logic gates [5]. However, conventional OB schemes suffer from relatively high thresholds due to the low nonlinear coefficients of the nonlinear materials employed, which hinders their practical application [6]. Consequently, the pursuit of low-threshold OB has remained a primary research direction in this field.

To reduce the OB threshold, current research mainly follows two pathways. One is to provide positive feedback for effective nonlinear enhancement through specific optical mechanisms [7], with typical structures including photonic crystal microcavities [8], prism-coupled configurations [9], and Fabry–Pérot resonators [10]. The other is to select materials with excellent nonlinear optical properties and incorporate them into specific structures to enhance the nonlinear effect, thereby achieving threshold reduction [11,12].

In terms of material selection, researchers have recently exploited various materials with strong nonlinear coefficients to reduce the OB threshold [13]. Among them, graphene has become a research hotspot in this field due to its superior third-order nonlinear optical properties, and low-threshold OB based on various composite structures of graphene has been successively reported [14,15]. For instance, Jiang et al. achieved local field enhancement via the excitation of optical Tamm states (OTSs) at the interface between monolayer graphene and a one-dimensional photonic crystal (1D-PhC), thereby obtaining low-threshold OB [16]. However, as a two-dimensional single-atomic-layer material, graphene imposes stringent requirements on fabrication processes, which limits the practical application of graphene-based OB schemes. Recently, a class of topological materials known as Weyl semimetal (WSM) has attracted extensive attention [17,18]. Previous studies have shown that, compared with conventional nonlinear materials, WSM possesses relatively large nonlinear coefficients in the terahertz regime [19,20], offering promising possibilities for low-threshold OB [21,22]. In 2023, researchers utilized the strong third-order nonlinearity of WSM and the local field enhancement of defect modes to achieve low-threshold OB in the terahertz regime in an asymmetric 1D-PhC structure containing WSM defect layers [23,24]. Therefore, WSM is expected to contribute to the realization of low-threshold nonlinear OB.

In this paper, we propose a layered structure composed of WSM and a multilayer 1D-PhC. By adjusting appropriate parameters, the structure achieves OB with a relatively low threshold within the photonic bandgap. In addition, we discuss the influence of other parameters on the OB threshold. We believe that this structure provides a new route toward tunable low-threshold OB and may find potential applications in all-optical devices.

## 2. Theoretical Model and Method

**Figure 1:**
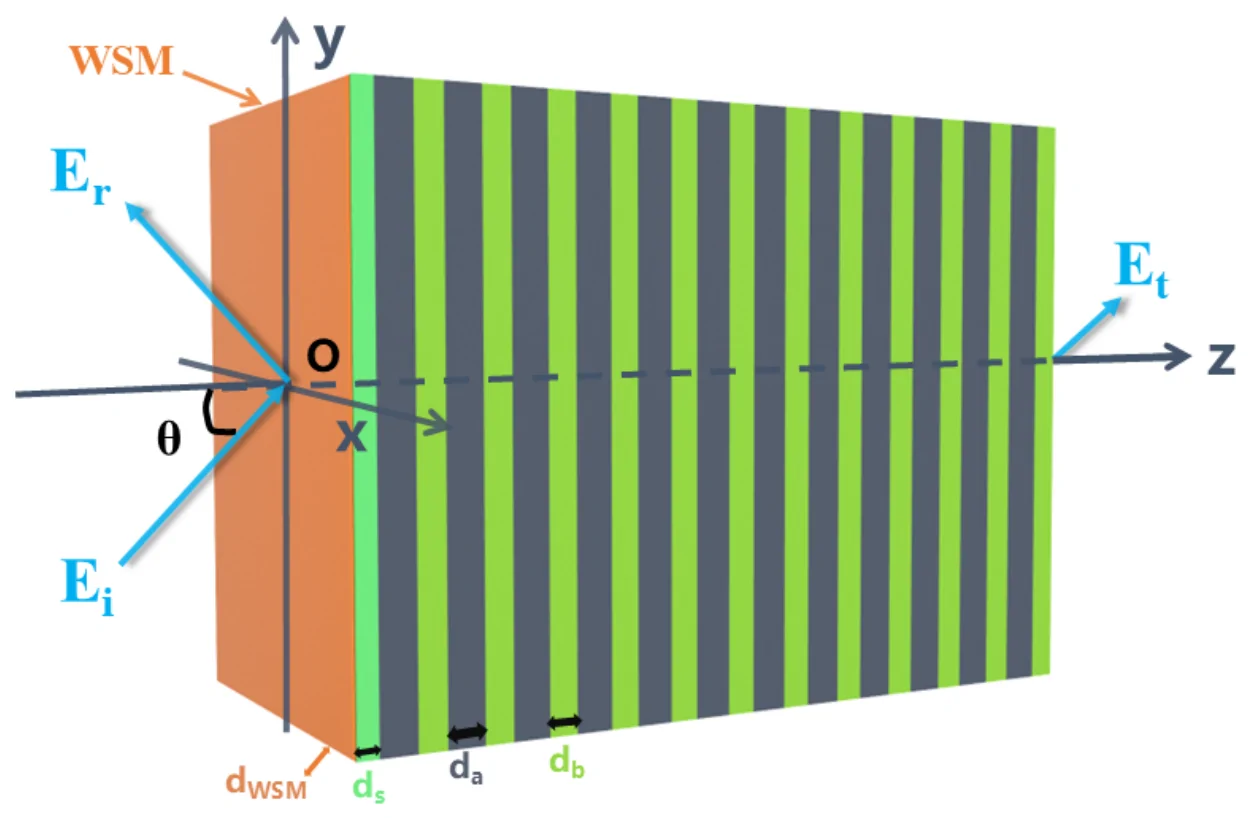
Schematic diagram of the multilayer structure based on WSM.

We consider a multilayer structure composed of WSM, a spacer layer, and a 1D-PhC, as shown in Figure 1. The functions of each module in constructing this structure are very clear: the nonlinear WSM layer is responsible for providing the basic conditions for the occurrence of the OB phenomenon in the entire structure; 1D-PhC construct bandgap features for the entire structure. The role of the spacer layer is reflected in two aspects: it can not only provide richer manipulation methods for the OB curve but also provide reserved structures for the optical modes that may be excited by the entire structure. In this structure, the incident light with an angle θ is launched from the left side, with the WSM layer located at the leftmost position. The incident electric field $E_{i}$, reflected electric field $E_{r}$, and transmitted electric field $E_{t}$ are indicated accordingly. The incident light propagates from the WSM side and exits from the rightmost side of the 1D-PhC. Essentially, Weyl semimetals (WSMs) are a general term for a class of materials. This type of material has similar material properties. Therefore, from a macro-perspective, the specific choice of materials is not the only consideration for whether the scheme can be implemented. However, considering the characteristics, preparation, cost, and other factors of various materials comprehensively in the scheme is also crucial for achieving a cost-effective OB solution. For now, we tend to choose WSM such as TaAs. Firstly, unlike the complex preparation process of two-dimensional materials such as graphene, the mentioned WSM can be synthesized naturally without the need for intricate and complex doping control, which also brings convenience to material preparation. Secondly, the second-order and third-order nonlinear optical properties of TaAs have been experimentally verified. In addition, the 1D-PhC is composed of alternating layers of medium A and medium B, and the number of periods is set to *N* = 12 in the following study. Poly(4-methyl-1-pentene) (TPX) is chosen as medium A with a refractive index of $n_{A}=1.46$, and medium B is SiO_2_ with a refractive index of $n_{B}=1.9$. In this work, the central frequency is set to $f_{c}=4.4\;\mathrm{THz}$. The spacer layer is located between the 1D-PhC and the WSM layer. Here, we use the same material as medium B for the spacer layer, which is SiO_2_ with ${n_{s}=n}_{B}=1.9$. If the thickness of the spacer layer is the same as that of medium B, then the structure is essentially a simple combination of WSM and 1D-PhC. However, if the thickness of the spacer layer is appropriately adjusted, it will provide richer control methods for the OB phenomenon. This is also what we hope to see. Therefore, here we set $d_{s}=\;0.75d_{B}$. The thicknesses of medium A and medium B are given by $d_{A}=\lambda_{c}/\left(4\times n_{A}\right),\;d_{B}=\lambda_{c}/\left(4\times n_{B}\right)$, respectively. The initial parameters of the WSM are set as: thickness $d_{\mathrm{WSM}\;}=\;1000\;\mathrm{nm}$ and Fermi energy $E_{F}$ = 5 meV.

The linear conductivity of WSM can be expressed as [23]

(1) $$\sigma_{\left(\omega \right)}^{\left(1\right)}=\frac{e^{2}v_{F}P_{F}^{2}g_{s}g_{\omega }}{6\pi^{2}\hbar^{3}(\gamma -i\omega )}$$

In the above expression, ω is the angular frequency of the incident light, and $v_{F}$ is the Fermi velocity, taken as 10^6^ m/s. $P_{F}$ is the magnitude of the electron momentum, given by $P_{F}=E_{F}/V_{F}$, where $E_{F}$ is the Fermi energy of WSM, set to 5 meV. ℏ is the reduced Planck constant, and ℏγ = 5 meV corresponds to the electron relaxation rate. In addition, in this work, the WSM is a non-magnetic WSM that preserves time-reversal symmetry and possesses two pairs of Weyl nodes. Accordingly, the spin degeneracy factor is $g_{s}$ = 2, and the Weyl node degeneracy factor is $g_{\omega }$ = 4. To simplify the model in this work, we consider WSM to be isotropic, and its linear permittivity can be expressed as [23]

(2) $$\varepsilon_{1}=\varepsilon_{b}+\frac{i4\pi \sigma^{\left(1\right)}}{\varepsilon_{0}\omega }$$

In Equation (2), $\varepsilon_{b}$ is the background permittivity, which takes a value of 10, and $\varepsilon_{0}$ is the vacuum permittivity. The third-order nonlinear permittivity of WSM can be expressed as [23]

(3) $$\sigma_{\left(\omega \right)}^{\left(3\right)}=\frac{e^{4}v_{F}g_{s}g_{\omega }}{15\pi^{2}\hbar^{3}(-i\omega +\gamma )}\left[\frac{\frac{1}{i\omega +\gamma }+\frac{1}{-i\omega +\gamma }}{\gamma }+\frac{1}{\left(-i2\omega +\gamma )(-i\omega +\gamma \right)}\right]$$

The third-order nonlinear susceptibility $\chi^{\left(3\right)}$ of WSM is given by

(4) $$\chi^{\left(3\right)}=\frac{i\sigma_{\left(\omega \right)}^{\left(3\right)}}{\varepsilon_{0}\omega }$$

Accordingly, the refractive index of WSM can be further expressed as

(5) $$n_{C}=\sqrt{\varepsilon_{1}+\chi^{\left(3\right)}|E_{Iside}{|}^{2}}$$

In Equation (5), $E_{Iside}$ is the electric field intensity on the incident side of the WSM, which consists of both the incident and reflected electric fields, namely, the incident electric field $E_{i}$ and the reflected electric field $E_{r}$ as shown in Figure 1.

Regarding the calculation of OB, generally speaking, different methods are used for different situations. For relatively simple few layer structures with nonlinear materials as substrates, the transmittance and reflectance of the entire structure can only be achieved by solving the nonlinear Maxwell’s equations [14]. For multilayer structures containing two-dimensional materials, the transmittance and reflectance of the entire structure can be achieved through the iteration of Maxwell’s equations and boundary conditions [19]. However, for non-layered structures such as metasurfaces, gratings, and other complex structures, the transmittance and reflectance of the entire structure need to be achieved through numerical simulation software [25]. Here, the transfer matrix method is employed to obtain the reflection and transmission coefficients of the whole structure, thereby establishing the relationship among the incident electric field, reflected electric field, and transmitted electric field. This is mainly because the current structure contains nonlinear bulk dielectric materials. We need to divide nonlinear bulk materials into many sublayers. This is essentially a numerical approximation [23,26].

Here, the stacking direction of the media is set along the z-axis, while the plane formed by the WSM layer, the spacer layer, and the 1D-PhC corresponds to the xy-plane. In this work, TE polarization is considered. With the WSM serving as the first layer, for the material in the *n*-th layer (*n* ≥ 2), the corresponding transfer matrix is given by

(6) $$M_{n}=\left(\begin{array}{cc} \cos(k_{nz}d_{n}) & -\frac{i}{\eta_{n}}\sin(k_{nz}d_{n})\\ -i\eta_{n}\sin(k_{nz}d_{n}) & \cos(k_{nz}d_{n}) \end{array}\right)$$

In the above transfer matrix, $k_{nz}$ denotes the wave vector of the *n*-th layer along the z-direction, and $d_{n}$ is the thickness of that layer. For TE polarization, $k_{nz}=(n_{n}\omega \;\cos\theta_{n})/c$, where $n_{n}$ is the refractive index of the *n*-th layer and $\theta_{n}$ is the refraction angle within that layer. $\eta_{n}$ is the optical admittance of the layer, given by $\eta_{n}=\sqrt{\varepsilon_{0}/\mu_{0}}n_{n}\;\cos(\theta_{n})$, where $\mu_{0}$ is the vacuum permeability and $\varepsilon_{0}$ is the vacuum permittivity.

Although WSM is a nonlinear material and its refractive index is not constant but depends on the local electric field intensity, it can be treated by dividing it into a sufficiently large number of slices. Since the WSM layer considered in this work is very thin, with reference to Equation (5), the electric field within each subdivided slice can be approximately regarded as a constant, provided that the number of divisions is sufficiently large [23].

Specifically, in this work, the transfer matrix $M_{Right}$ corresponding to the spacer layer and the photonic crystal on the right side of the WSM is first obtained as $M_{Right}=M_{s}(M_{A}M_{B}{)}^{12}$. Then, the electric field at the interface between the WSM and the spacer layer satisfies the following relation:

(7) $$E_{Iside}=M_{Right}(1,1)*E_{t}\;+\;M_{Right}(1,2)*E_{t}*\eta_{N}$$

In Equation (7), $E_{Iside}$ is the electric field on the right side of the WSM. Since the WSM is divided into a sufficiently large number of sublayers, as mentioned above, the electric field near each sublayer can be approximately treated as a constant. The electric field on the right side of the WSM is taken as the representative value of the field near the WSM. Therefore, here $E_{Iside}$ is equivalent to the electric field at the incident-side interface of the WSM layer. $E_{t}$ is the transmitted electric field of the structure corresponding to $M_{Right}$, i.e., the composite structure consisting of the spacer layer and the 1D-PhC with a period of 12. $\eta_{N}$ is the optical admittance of the medium on the transmission. In this work, for the structure composed of the WSM, the spacer layer, and 1D-PhC, both the incident-side and transmission-side media are vacuum.

Since the WSM is divided into *n* sublayers along the direction perpendicular to the incident light, the transfer matrix corresponding to the *n*-th sublayer can be expressed as

(8) $$M_{nW}=\left(\begin{array}{cc} \cos(k_{nWz}d_{nW}) & -\frac{i}{\eta_{n}}\sin(k_{nWz}d_{nW})\\ -i\eta_{n}\sin(k_{nWz}d_{nW}) & \cos(k_{nWz}d_{nW}) \end{array}\right)$$

In Equation (8), $k_{nWz}$ is the wave vector of the *n*-th WSM sublayer along the direction perpendicular to the incident light, $d_{nW}$ is the thickness of that sublayer, and $\eta_{N}$ is the optical admittance of the *n*-th WSM sublayer. Each subdivided WSM sublayer can be treated in a similar manner. Thus, the transfer matrix corresponding to the entire multilayer WSM structure can be obtained as

(9) $$\left[\begin{array}{c} E_{1}\\ H_{1} \end{array}\right]=M_{1}M_{2}M_{3}\ldots M_{nW-2}M_{nW-1}M_{nW}M_{Right}\left[\begin{array}{c} E_{n+1}\\ H_{n+1} \end{array}\right]$$

where $E_{1}$ and $H_{1}$ are the electric and magnetic fields at the incident-side interface of the WSM, corresponding to the incident-side fields of the first subdivided WSM sublayer. $E_{n\;+\;1}$ and $H_{n\;+\;1}$ are the electric and magnetic fields on the transmission side of the entire structure, i.e., the fields at the rightmost side of the photonic crystal.

The transfer matrix of the entire WSM structure can be expressed as

(10) $$M_{WSM}=M_{1}M_{2}M_{3}M_{4}\cdots M_{nW-2}M_{nW-1}M_{nW}$$

where $M_{1}$, $\;M_{2}$, …, $\;M_{nW}$ denote the transfer matrices of each subdivided WSM sublayer, respectively.

At this point, the overall transfer matrix of the entire structure consisting of the WSM, the spacer layer, and the 1D-PhC with a period of 12 is given by

(11) $$M=M_{WSM}M_{Right}=M_{WSM}M_{s}(M_{A}M_{B}{)}^{12}$$

Next, the transmission coefficient of the entire structure can be obtained by using the total transfer matrix:

(12) $$t=2\eta_{0}/(M_{11}\eta_{0}\;+\;M_{12}\eta_{0}\eta_{N}\;+\;M_{21}\;+\;M_{22}\eta_{N})$$

And the corresponding reflection coefficient of the entire structure can be obtained as

(13) $$r=\frac{M_{11}\eta_{0}+M_{12}\eta_{0}\eta_{N}-M_{21}-M_{22}\eta_{N}}{M_{11}\eta_{0}+M_{12}\eta_{0}\eta_{N}+M_{21}+M_{22}\eta_{N}}$$

According to the transmission and reflection coefficients of the entire structure, the relationship between the incident electric field and the transmitted electric field can be obtained as

(14) $$\left|E_{t}|=|E_{i}|\times |t\right|$$

Meanwhile, the relationship between the incident electric field and the reflected electric field can also be obtained as

(15) $$\left|E_{r}|=|E_{i}|\times |r\right|$$

It is necessary to clarify that we have adopted a relatively idealized model in the schematic diagram. That is, we set both sides of the entire structure as air. However, if one intends to manufacture the proposed structure, a realistic substrate must be considered. In fact, this structure is a layered structure that does not require high processing technology. In addition, in the following calculations, we only consider the reflective OB phenomenon. Hence, in practical situations, we only need to add a real substrate to the right end of the schematic diagram shown in Figure 1. The choice of this substrate can also be diversified: it can be the same material as medium A or B, or a separate substrate material (such as germanium). The introduction of the substrate may slightly adjust the OB threshold. However, it will not affect the core features of the entire structure. Therefore, we will not discuss further here.

## 3. Results and Discussion

**Figure 2:**
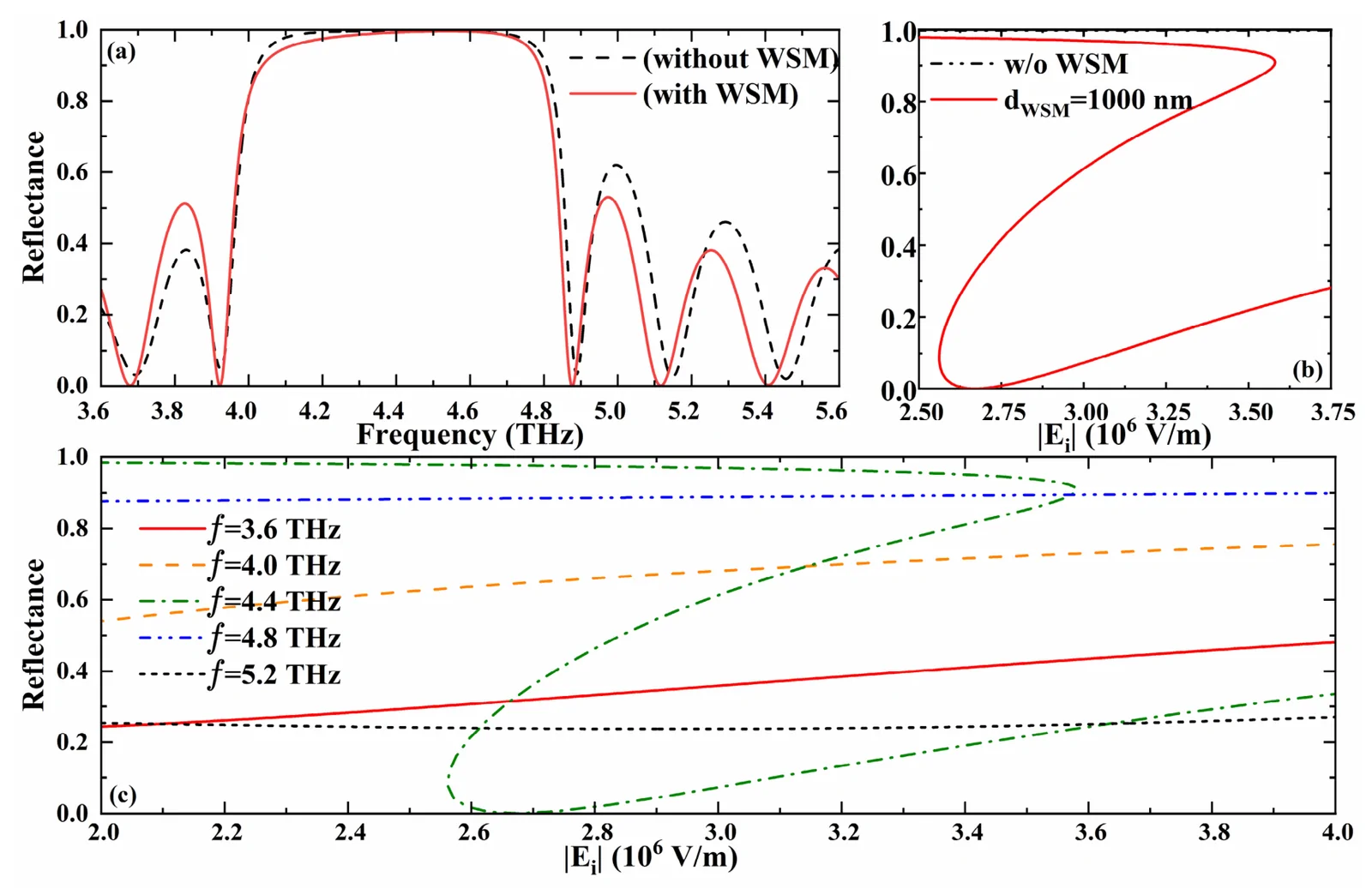
(**a**) Reflectance as a function of incident light frequency for the whole structure with and without WSM, where the WSM thickness is 1000 nm; (**b**) reflectance versus incident electric field with and without WSM; (**c**) reflectance as a function of incident electric field for the structure at different incident light frequencies.

In this section, we will focus on discussing the OB phenomenon in the photonic bandgap. From Figure 2a, the reflectance of the WSM-spacer-1D-PhC composite structure as a function of the incident light frequency can be observed. The figure shows that the photonic bandgap of the structure is located in the range of 4.0 THz to 4.8 THz, within which the reflectance exceeds 0.98 and reaches nearly 1.00 at 4.4 THz; outside the bandgap, the reflectance drops rapidly to values between 0.25 and 0.40. Figure 2b presents the relationship between the incident electric field and the reflectance at a frequency of 4.4 THz, with and without the WSM layer. It is clearly observed that in the absence of the WSM layer, the reflectance remains nearly constant as the incident electric field increases, appearing as a flat straight line, and no OB phenomenon is observed. In contrast, when the WSM layer with a thickness of 1000 nm is introduced, the reflectance–incident electric field curve displays a distinct S-shaped hysteresis loop, indicating the emergence of OB. Figure 2c presents the reflectance as a function of the incident electric field at five different frequencies. At 3.6 THz, 4.0 THz, 4.8 THz, and 5.2 THz, the reflectance remains nearly stable with increasing electric field, staying around 0.25, 0.98, 0.90, and 0.90, respectively, and no OB is observed within the considered range of the incident electric field. However, at 4.4 THz, the reflectance jumps from approximately 0.9 to approximately 0.2 as the incident electric field increases, exhibiting distinct bistable behavior. From the above observations, it is evident that this scheme possesses wavelength-selective characteristics. In conventional wavelength-selective switches, the filtering and switching functions are implemented by cascaded discrete devices. In the present structure, however, frequency identification and optical intensity control are no longer two separate processes but are accomplished synergistically within a single device by the photonic crystal, the spacer layer, and the WSM. This feature is of great significance for optical switching applications and thus provides possibilities for the design of corresponding terahertz devices.

**Figure 3:**
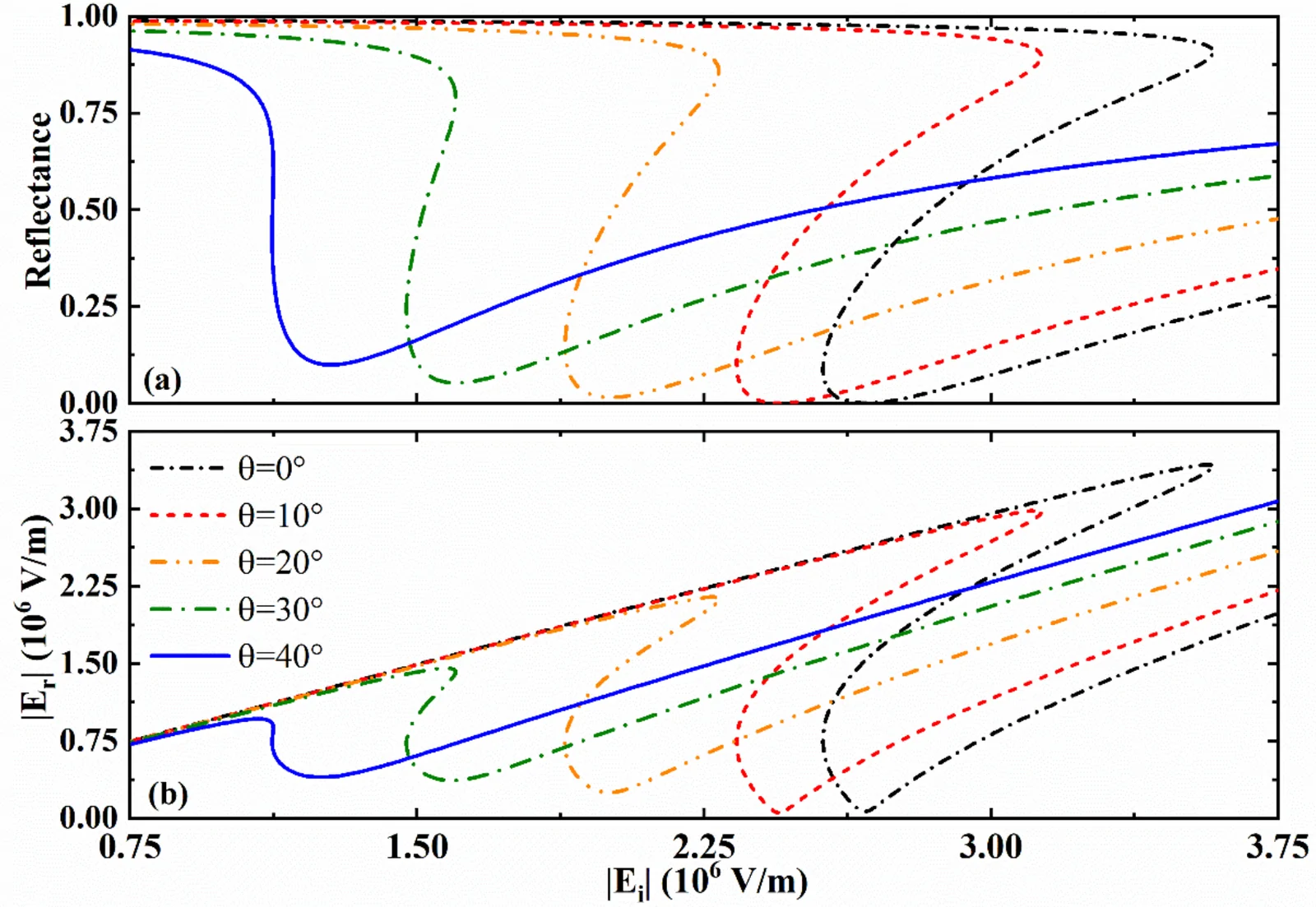
(**a**) Reflectance and (**b**) reflected electric field as functions of the incident electric field at different incident angles. Here, the incident light frequency is fixed at 4.4 THz, the WSM thickness is 1000 nm, and the Fermi energy is 5 meV.

Next, we discuss the influence of the incident angle on the OB threshold when the incident frequency, WSM thickness, and Fermi energy are fixed, as shown in Figure 3a,b. At an incident angle of 0°, a distinct OB curve is observed, where the upper threshold is $|E_{i}{|}_{up}=2.56\times 10^{6}\;$ V/m, the lower threshold is $|E_{i}{|}_{down}=3.58\times 10^{6}\;$ V/m, and the corresponding hysteresis width is $\Delta \left|E_{i}\right|=1.02\times 10^{6}$ V/m. As the incident angle increases, both the upper and lower thresholds decrease, and the hysteresis width gradually narrows. When the incident angle exceeds 40°, the OB phenomenon disappears. The changes in incident angle are essentially regulating the phase part $k_{nWz}d_{nW}$ in the transmission matrix. We know that $k_{nWz}$ = $n_{C}$ cos θ/c. It is not difficult to see that an increase in θ will result in a decrease in $k_{nWz}$. Based on the calculation rule of the transmission matrix, the reduction in the phase part will cause a trend adjustment in the entire M matrix. That is also why we can see the changes in these trends in Figure 3. Although our goal is to reduce the threshold, it is also necessary to ensure the existence of the OB phenomenon. It is found that the incident angle plays a significant role in the OB behavior, indicating that tuning the incident angle is a viable approach for modulating OB.

**Figure 4:**
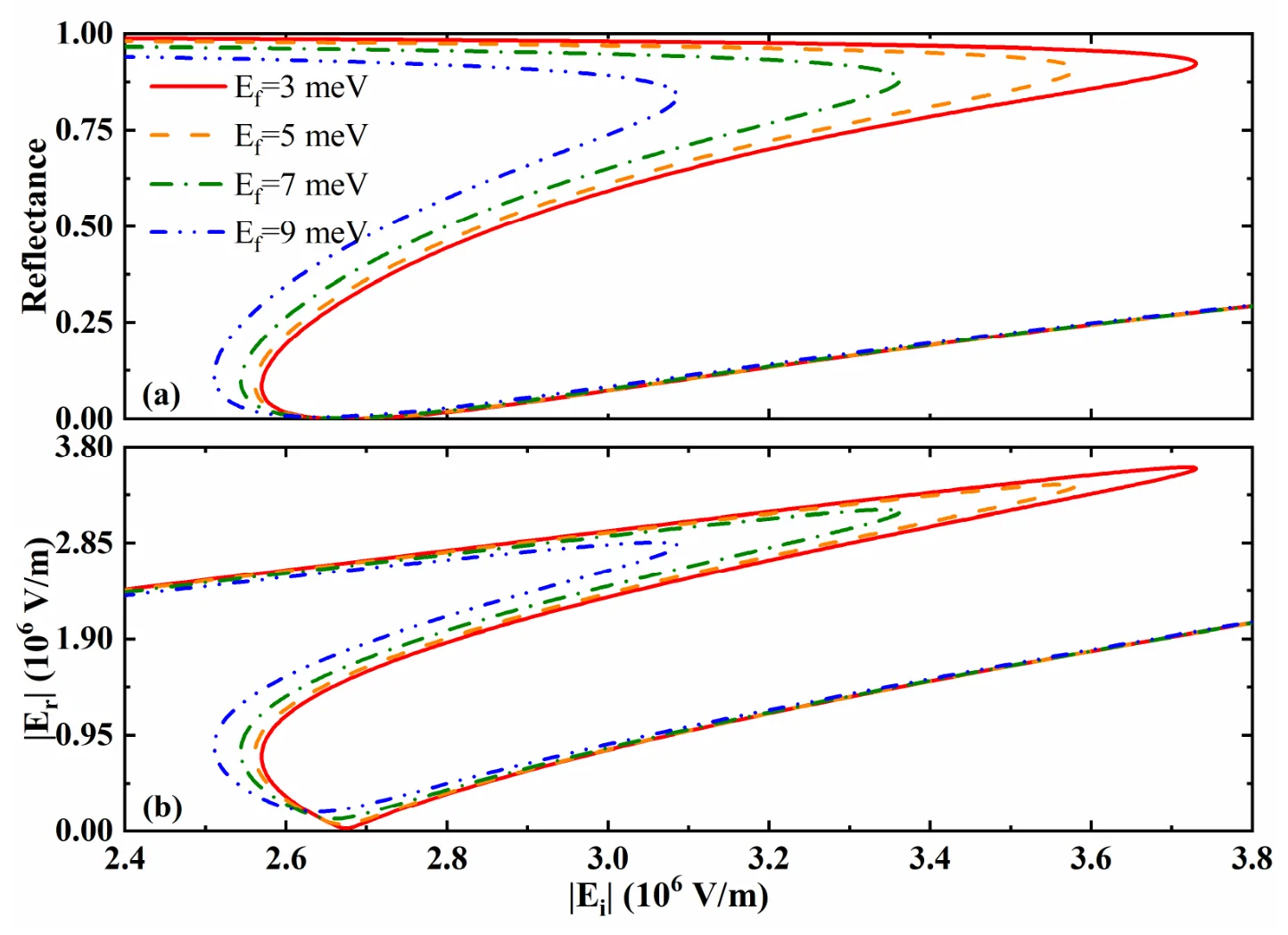
(**a**) Reflectance and (**b**) reflected electric field as functions of the incident electric field for different Fermi energies.

After discussing the influence of the incident angle on OB, we proceed to examine the effect of Fermi energy on OB with the incident angle fixed at 0° and other parameters unchanged, as shown in Figure 4. The regulatory characteristics of Fermi energy are that although the Fermi energy has a certain regulatory effect on the threshold and threshold width, the sensitivity of regulation is relatively limited. This is because the changes in Fermi energy are mainly concentrated on relatively small variations in nonlinear WSM. It can be observed from Figure 4 that as the Fermi energy increases from 3 meV to 9 meV, the OB phenomenon persists, and both the upper and lower thresholds decrease with increasing Fermi energy. Taking the Fermi energy of 3 meV as an example, the upper threshold is $|E_{i}{|}_{up}=2.57\times 10^{6}\;$ V/m, the lower threshold is $|E_{i}{|}_{down}=3.72\times 10^{6\;}\;$ V/m, and the corresponding hysteresis width is $\Delta \left|E_{i}\right|=1.15\times 10^{6}\;$ V/m. When the Fermi energy increases to 9 meV, the upper threshold becomes $|E_{i}{|}_{up}=2.51\times 10^{6}\;$ V/m, the lower threshold becomes $|E_{i}{|}_{down}=3.08\times 10^{6}\;$ V/m, and the hysteresis width is $\Delta \left|E_{i}\right|=0.57\times 10^{6}\;$ V/m. It is not difficult to see that the increase in Fermi energy not only slightly lowers the threshold of OB, but also significantly reduces the width of the threshold. The main reason for the slight decrease in threshold can be found in the linear conductivity (Equation (1)) and refractive index expression (Equation (5)) of WSM. It is not difficult to see from the expression that an increase in Fermi energy can significantly increase the linear conductivity of WSM, thereby enhancing its refractive index and nonlinear coefficient to a certain extent, which has a positive effect on reducing the threshold under the same conditions. However, the increase in Fermi energy is not friendly to the threshold width. This can be addressed by similar structures based on graphene (WSM has many similarities with graphene in terms of linear/nonlinear conductivity expressions). In Xu et al.’s work, they quantitatively derived the relationship expression between threshold width and conductivity based on approximation [27]: $\Delta Y=-\frac{4}{27{\left|t_{phc1}\right|}^{2}{\left|t_{phc2}\right|}^{2}}\frac{{\left(\Psi Z\sigma_{0}\;+\;\Gamma \right)}^{3}}{\Psi Z\sigma_{3}}$. Equations (1) and (3) clearly demonstrate that an increase in Fermi energy significantly enhances linear conductivity but has little effect on nonlinear conductivity. Referring to the threshold width expression, it is not difficult to find that the increase in Fermi energy has a significant decreasing relationship with the threshold width. This also explains why the regulatory pattern in Figure 4 appears.

**Figure 5:**
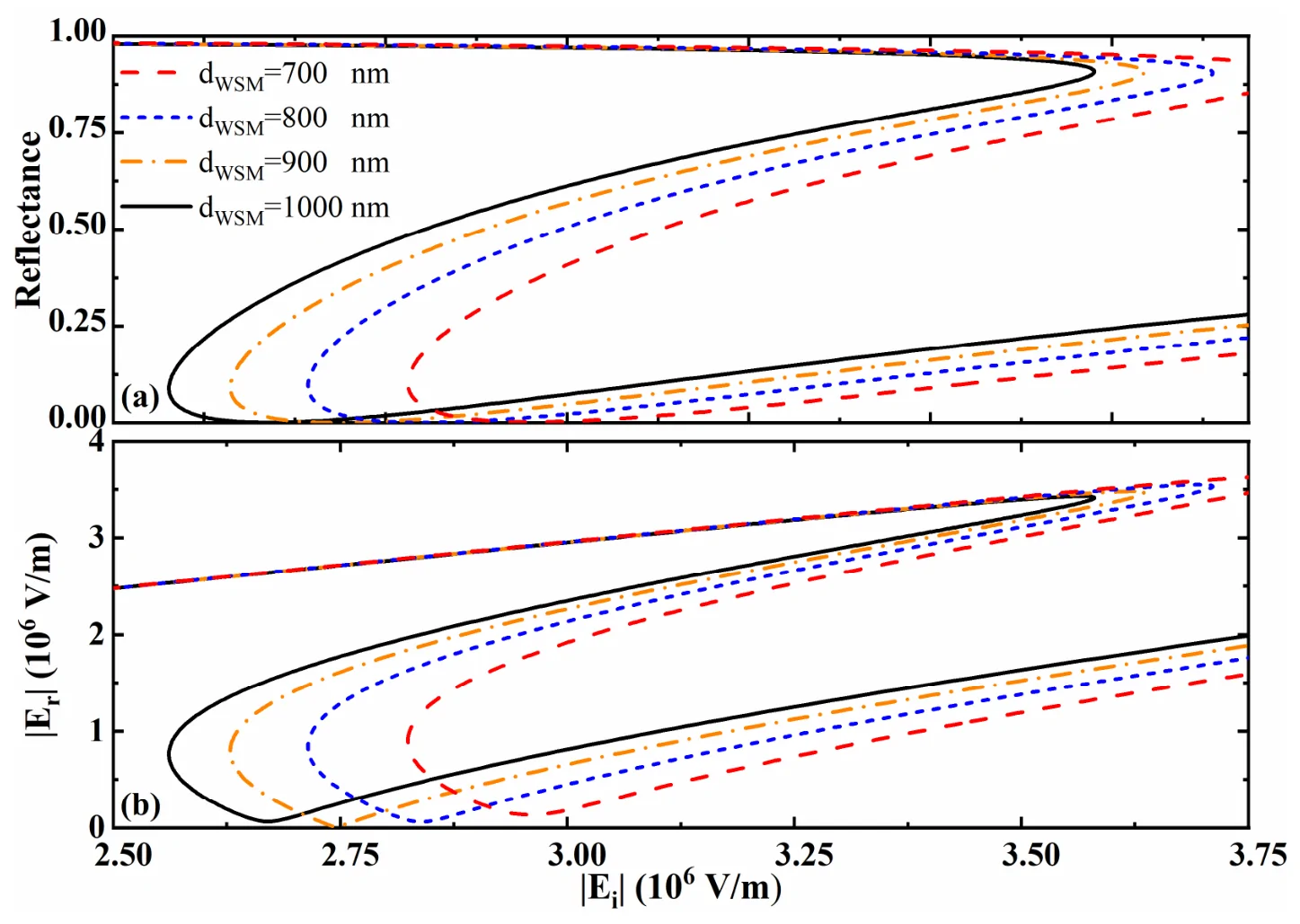
(**a**) Reflectance and (**b**) reflected electric field as functions of the incident electric field for different WSM thicknesses.

We also discuss the influence of WSM thickness on OB when the incident angle, frequency, and Fermi energy of WSM are fixed, as shown in Figure 5. With other parameters unchanged, only the WSM thickness is varied to examine its effect on OB. It can be observed that the effect of WSM thickness variation on OB exhibits a certain similarity to that of Fermi energy variation. Taking the WSM thickness of 800 nm as an example, the upper threshold is $|E_{i}{|}_{up}=2.67\times 10^{6}\;$ V/m, the lower threshold is $|E_{i}{|}_{down}=3.71\times 10^{6}\;$ V/m, and the corresponding hysteresis width is $\Delta \left|E_{i}\right|=1.04\times 10^{6}\;$ V/m. When the WSM thickness increases to 1000 nm, the upper threshold becomes $|E_{i}{|}_{up}=2.56\times 10^{6}\;$ V/m, the lower threshold becomes $|E_{i}{|}_{down}=3.58\times 10^{6}\;$ V/m, and the hysteresis width is $\Delta \left|E_{i}\right|=1.02\times 10^{6}\;$ V/m. By comparing the OB characteristics at WSM thicknesses of 800 nm and 1000 nm, it is found that even when the WSM thickness changes considerably, the hysteresis width varies only slightly, and the threshold magnitudes also exhibit little change. Although the hysteresis width changes to some extent with WSM thickness, the overall variation is relatively small. This indicates that OB devices based on such structures are relatively stable, and their performance is not significantly affected by considerable variations in the WSM thickness. The understanding of the regulation law of WSM thickness is relatively simple. On the premise of keeping the number of sublayers unchanged, an increase in WSM thickness will result in a slight increase in the thickness of a single sublayer compared to before. We use recursion and iteration from right to left when calculating the electric field distribution. This algorithm will slow down the attenuation of the electric field in each sublayer as the thickness of WSM increases, resulting in a slight increase in the equivalent nonlinear coefficient of each sublayer. This also creates conditions for lowering the threshold. However, it should be noted that the relatively limited increase in the thickness of the sublayers also results in a relatively limited decrease in the threshold. This also explains the trend we see in Figure 5.

**Figure 6:**
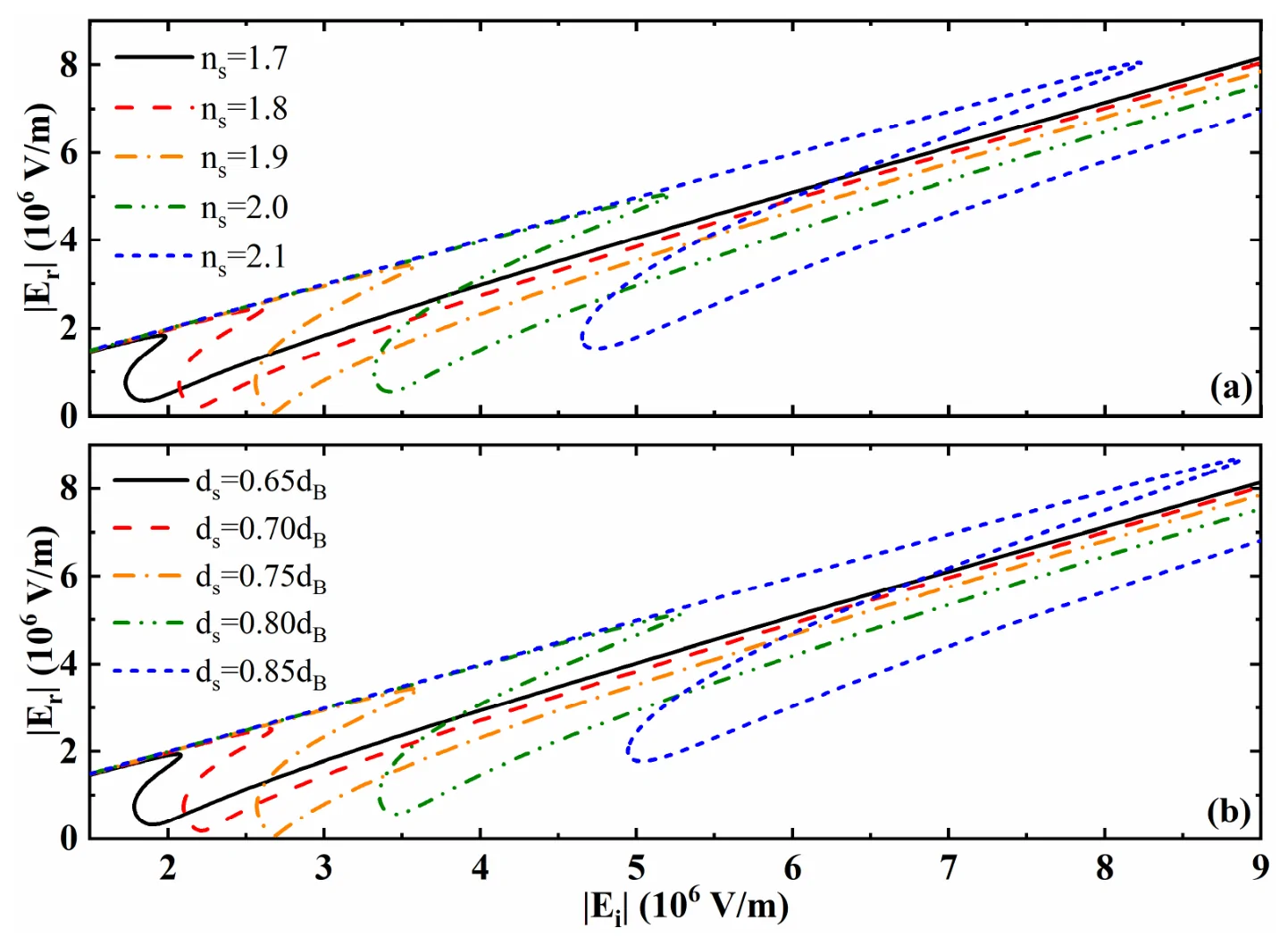
Reflected electric field as a function of the incident electric field for the spacer layer with (**a**) different refractive indices $n_{s}$ and (**b**) different thicknesses $d_{s}$.

Finally, we discuss the influence of the spacer layer parameters on the OB phenomenon. When the refractive index of the spacer layer is varied, its thickness is fixed at $d_{s}=0.75d_{B}$; when the thickness is changed, the refractive index is fixed at $n_{s}$ = 1.9. Figure 6a shows the variation in OB characteristics with the refractive index of the spacer layer. We found that the decrease in $n_{s}$ significantly lowers the threshold of OB. However, this does not mean that the $n_{s}$ value can be infinitely reduced in order to obtain a lower threshold. Firstly, $n_{s}$ represents the refractive index of the spacer layer, which has practical significance. Its value must normally be greater than 1. This sets a limit for the continuous decrease of $n_{s}$. Secondly, although the reduction of $n_{s}$ can continuously lower the threshold of OB, it also comes with the cost of decreasing the threshold width. This means that if we blindly reduce $n_{s}$, the hysteresis curve will eventually disappear and the optical bistable phenomenon will be lost. The effect of increasing the spacer layer thickness on OB characteristics is similar to that of the refractive index, as shown in Figure 6b. When $d_{s}=0.85d_{B}$, the upper threshold is $|E_{i}{|}_{up}=4.95\times 10^{6}\;$ V/m, the lower threshold is $|E_{i}{|}_{down}=8.87\times 10^{6}\;$ V/m, and the hysteresis width is $\Delta \left|E_{i}\right|=3.92\times 10^{6}\;$ V/m. When $d_{s}=0.65d_{B}$, the upper threshold is $|E_{i}{|}_{up}=1.78\times 10^{6}\;$ V/m, the lower threshold is $|E_{i}{|}_{down}=2.08\times 10^{6}\;$ V/m, and the hysteresis width is $\Delta \left|E_{i}\right|=3\times 10^{5}\;$ V/m. It can be seen that as $d_{s}$ decreases, the hysteresis width gradually narrows, and when $d_{s}$ becomes too small, the OB phenomenon disappears. This result indicates that the spacer layer parameters play an important modulating role in the nonlinear optical response. The effect of the refractive index and thickness of the spacer layer on OB is essentially similar to the effect of the incident angle in Figure 3. The changes in refractive index and thickness of the spacer layer are essentially regulating the phase part $k_{nWz}d_{nW}$ in the transmission matrix.

It is not difficult to see that a decrease in $n_{s}$ will result in a decrease in $k_{nWz}$. These are consistent with the effect of $k_{nWz}d_{nW}$ caused by the reduction of $d_{nW}$. The reduction in the phase part will also cause a trend adjustment in the entire M matrix. That is also why we can see the changes in these trends in Figure 6. Hence, selecting appropriate refractive index and thickness of the spacer layer not only allows tuning of the OB threshold range but also affects the hysteresis width, thereby significantly influencing the stability and operating characteristics of OB devices. Therefore, when designing nonlinear optical devices based on similar structures, the spacer layer parameters should be comprehensively considered in order to achieve stable and tunable OB. We have also compared our proposed structure with other reported papers and found that our structure has lower threshold and simple structure, as shown in Table 1.

## 4. Conclusions

In this paper, we investigate tunable OB in the composite structure of WSM and layered media. A multilayer structure model consisting of a WSM layer, a spacer layer, and 1D-PhC is first constructed. By exploiting the significant third-order nonlinearity of WSM in the terahertz regime, the feasibility of achieving low-threshold OB in this structure is studied. The results show that the introduction of WSM leads to a notable modification of the reflectance spectrum within a specific frequency range, which provides the necessary conditions for the emergence of OB. After parameter optimization, low-threshold tunable OB is achieved in the terahertz regime, with an incident electric field threshold reaching 10^6^ V/m. By adjusting parameters such as the incident angle, the thickness of the WSM layer, and the Fermi energy, the bistable threshold can be effectively modulated, demonstrating the frequency-tunable characteristics of the structure within the bandgap. These results provide a new route for designing low-threshold and high-stability OB devices and may find potential applications in the field of nonlinear optical devices.

## Figures and Tables

**Table 1:** Comparison between different optical bistable schemes.

Ref.	Mechanism	Structure	Threshold	Process Requirements	Frequency Range
[16]	Optical Tamm State	Graphene-Bragg reflector structure	4.3 × 10^6^ V/m	High	THz
[23]	Defect mode	Asymmetrical 1D-PhCs	49.39 MW/m^2^	Moderate	THz
[27]	Cavity Resonance	Photonic crystal cavity	2.5 × 10^5^ V/m	High	THz
[28]	Fano resonance	1D photonic crystal Fano resonance heterostructures	4.7 × 10^7^ V/m	High	Near Infrared
[7]	Conjugate Topological Edge State	Conjugate Topological Photonic Crystal	2 µW/m	Moderate	Near Infrared
[29]	Cavity resonance	Photonic Crystals Cavity	1.0 × 10^6^ V/m	High	THz
[30]	SPP	Otto Configuration	3.0 × 10^6^ V/m	High	THz
This work	Photonic crystal bandgap	WSM-1D-PhC	1.0 × 10^6^ V/m	Simple	THz

## Data Availability

The data presented in this study are available on request from the corresponding author.
